# Role of Language-Derived Semantics in Knowledge Distillation on Wearable Sensor Data

**DOI:** 10.3390/biomimetics11100729

**Published:** 2026-10-12

**Authors:** Min Chan Kang, Jae Chan Jeong, Pavan Turaga, Eun Som Jeon

**Affiliations:** 1 Department of Computer Science and Engineering, Seoul National University of Science and Technology, Seoul 01811, Republic of Korea; smartkmc02@seoultech.ac.kr (M.C.K.); wocks1626@seoultech.ac.kr (J.C.J.); 2 Geometric Media Lab, The GAME School, Arizona State University, Tempe, AZ 85281, USA; pturaga@asu.edu

**Keywords:** knowledge distillation, human activity recognition, wearable sensors, language-derived semantics, semantic guidance, multimodal learning

## Abstract

Wearable sensor-based human activity recognition (HAR) has been widely studied for understanding human activities from time-series data. However, wearable sensor data exhibit substantial inter-individual variations, while the signal differences between activities can be relatively subtle. These characteristics make it challenging to learn effective representations for activity recognition. Considering these limitations, human cognition provides a useful motivation, as raw signals are interpreted together with prior knowledge and contextual expectations, suggesting that language-derived semantics can complement the information contained in time-series data. However, incorporating such semantic information into wearable systems requires careful consideration of the computational and memory constraints of edge devices. In this regard, knowledge distillation (KD) enables richer information to be exploited during training while retaining a compact student model for inference. Nevertheless, how language-derived semantics contribute to KD on wearable sensor data remains underexplored. In this study, we systematically investigate how textual semantics can be incorporated into KD through a Semantic Guidance Module (SGM), considering three different strategies: teacher-side (T-SG), student-side (S-SG), and both-side (B-SG). We examine these strategies through various evaluations, including analyses of teacher–student architectures, prompt types, and window lengths. Our findings demonstrate that language-derived semantics provide complementary knowledge for student learning and offer insights into how semantic information can be effectively incorporated into KD for wearable sensor-based HAR.

## 1. Introduction

Wearable sensor-based human activity recognition (HAR) has been applied to a wide range of real-world applications, including healthcare [1], smart home monitoring [2], abnormal behavior detection for elderly care [3], and human-computer interaction [4,5]. With these growing demands, deep learning methods have been widely developed to achieve more accurate and reliable activity recognition [4,6]. However, wearable sensor data present challenges for analysis due to individual variations, sensor noise, and differences in data acquisition conditions [7,8,9,10]. These factors make it challenging for models to learn effective representations and achieve high recognition performance.

In human cognition to address these limitations [11,12], when people interpret information, whether auditory, visual, or physical, they rarely rely on raw data alone. Instead, such data are interpreted in conjunction with contextual expectations and prior semantic knowledge [11,12]. In this way, language can provide additional semantic information associated with human actions [13,14], which may help reduce representation ambiguity.

Motivated by this, language-derived semantic information can be used to enrich sensor representations for HAR. However, directly using such semantic information in HAR requires additional resources for processing textual information, increasing computational and memory requirements.

This poses a practical challenge for wearable edge devices, which typically operate with limited computational, memory, and power resources while requiring low-latency processing of continuous sensor streams [4,15]. These considerations highlight the need for a learning framework that can exploit richer semantic information while maintaining a compact model suitable for resource-constrained deployment.

Knowledge distillation (KD) has been widely utilized as a promising method to develop a compact model (student) by leveraging knowledge from a high-capacity model (teacher) [16,17]. In conventional KD, the student learns to mimic the predictive behavior of the teacher while maintaining a lightweight architecture for inference. More broadly, various knowledge transfer frameworks have been explored, including feature-based distillation [18], relational distillation [19], multi-teacher frameworks [20,21], and multimodal KD [22,23]. These advances suggest that KD can support not only model compression but also the transfer of complementary information, which provides a basis for incorporating language-derived semantics into the distillation process. However, how language-derived semantic information affects KD for wearable sensor-based HAR remains largely unexplored.

To investigate this effect, we design a Semantic Guidance Module (SGM)-based KD framework that incorporates activity-level, language-derived semantics into latent sensor representations. Specifically, we consider three distinct semantic conditioning strategies that differ in where semantic conditioning is applied within the KD framework: (1) Teacher-side SG (T-SG), which applies semantic conditioning to the teacher; (2) Student-side SG (S-SG), which applies semantic conditioning to the student; and (3) Both-side SG (B-SG), which applies semantic conditioning to both the teacher and student. All strategies use the same textual semantics but differ in where and how semantic conditioning is incorporated into the KD process. With these strategies, we comprehensively analyze their effectiveness across various experiments, including prompt type, window length, semantic density, distillation temperature, and model reliability.

Our key findings and contributions are summarized as follows:

- We design an SGM-based KD framework that incorporates textual semantics of activity into latent sensor representations to investigate how semantic information affects KD.
- We investigate three semantic conditioning strategies, T-SG, S-SG, and B-SG, to examine which side of the KD framework—the teacher, the student, or both—benefits most from semantic conditioning.
- We conduct empirical experiments to analyze the effects of different prompt types, such as keywords and sentences.
- We further analyze the strategies with diverse perspectives, including their sensitivity to window length, semantic density, distillation temperature, and model reliability.

## 2. Background

### 2.1. Representation Learning in Wearable Sensor HAR

Wearable sensor-based human activity recognition aims to analyze multivariate time-series signals acquired from body-worn sensors such as accelerometers and gyroscopes [4]. In deep learning-based HAR, sensor signals are transformed through a feature encoder into latent representations that capture activity-related characteristics of the input [4,8]. These latent representations are then passed to a classifier to predict the corresponding activity class.

Previous studies have explored network architectures designed to learn richer representations and improve data analysis performance. For example, convolutional and recurrent layers have been combined to capture local features and temporal dependencies [24], and attention-based modules have been incorporated to selectively capture informative sensor features and temporal information [25]. In general, increasingly complex architectures with stacked and specialized layers have been investigated to further enhance representation learning [26]. Although adding such layers and modules can improve model performance, it inevitably increases model complexity and computational costs [26], making these approaches less suitable for resource-constrained devices commonly used for wearable sensor data analysis.

### 2.2. Knowledge Distillation

Knowledge distillation (KD) provides a teacher–student learning paradigm for transferring predictive information from a high-capacity model to a more compact model. The underlying idea of transferring the predictive behavior of large models to smaller models was explored in a previous study [27], and Hinton et al. [16] later formalized and popularized this approach as KD using temperature-scaled soft targets. Unlike standard supervised training that relies only on ground-truth labels, conventional KD additionally uses the teacher’s output distribution as a learning signal for the student. Accordingly, the conventional KD objective combines label-based supervision with teacher-based distillation as follows:

(1) $$L=\left(1-\lambda \right)L_{CE}+\lambda L_{KD},$$

where 0<λ<1 is a hyperparameter that determines the relative weighting between the cross-entropy loss $L_{CE}$ and the distillation loss $L_{KD}$. The cross-entropy loss is computed from the student prediction and the corresponding ground-truth label:

(2) $$L_{CE}=Q\left(\sigma \left(o_{S}\right),y\right),$$

where Q(·) and σ(·) denote the cross-entropy and softmax functions, respectively, while $o_{S}$ and *y* represent the student logits and the ground-truth label. For KD, the output distributions of the teacher and student are compared after temperature scaling. The corresponding distillation loss is formulated as:

(3) $$L_{KD}=\tau^{2}KL\left(\sigma \left(o_{T}/\tau \right),\sigma \left(o_{S}/\tau \right)\right),$$

where $o_{T}$ denotes the teacher logits, and KL(·) denotes the Kullback–Leibler divergence between the temperature-scaled teacher and student distributions. The temperature parameter τ controls the degree of smoothing applied to the output distributions. As τ increases, probability mass is distributed more broadly across classes, revealing the teacher’s relative preferences for non-target classes and providing additional information for student learning [16,17].

Beyond conventional logit-based KD, various approaches have been developed to transfer richer forms of knowledge. These include feature-based and relational distillation methods, which use intermediate representations or feature relationships [18,19], as well as multi-teacher and multimodal KD, which exploit complementary knowledge from multiple models or modalities [20,21,22,23]. These approaches enable the student to leverage richer knowledge during learning.

### 2.3. Language-Guided Representation Learning

Language has been increasingly used as an additional source of semantic information for representation learning. In computer vision, language-guided learning enables visual models to incorporate semantic concepts expressed through textual information and has contributed to advances in tasks such as image recognition, segmentation, and generation [28,29,30,31]. These studies demonstrate the potential of language-derived information to complement representations learned from visual observations and provide additional semantic cues for downstream tasks.

Building on these advances, language-based approaches have recently been explored in time-series and wearable sensor applications [32]. In wearable HAR, textual information has been used to provide activity-related semantic information for sensor-based representation learning [33,34]. These developments reflect a growing interest in leveraging language-derived knowledge to complement information available from sensor measurements.

Language-derived semantic information has also been explored in KD. Prior studies have incorporated textual representations into KD to provide additional semantic guidance during knowledge transfer [35,36]. However, prior studies have primarily explored language-guided distillation in visual domains, leaving the role of language-derived semantic information in knowledge distillation (KD) for wearable sensor-based human activity recognition (HAR) relatively underexplored. In particular, the impact of incorporating semantic guidance at different stages of the KD framework on the distillation of wearable sensor representations remains insufficiently investigated.

## 3. Proposed Method

In this section, we describe the methodology used to incorporate language-derived semantic information of activity into KD for wearable sensor-based HAR. We define the sensor and text representations and then introduce the Semantic Guidance Module (SGM) used to incorporate textual semantics into latent sensor representations. We present the three semantic conditioning strategies and their corresponding training procedures.

### 3.1. Sensor and Text Representations

Let $x\in R^{L\times C}$ denote a segmented multichannel time-series sample with *L* time steps and *C* sensor channels. The teacher and student encoders, denoted by $f_{T}$ and $f_{S}$, independently transform the same input into their respective latent representations, $z_{T}=f_{T}\left(x\right)\in R^{d_{T}}$ and $z_{S}=f_{S}\left(x\right)\in R^{d_{S}}$, where $d_{T}$ and $d_{S}$ denote the dimensions of the teacher and student latent representations, respectively. These representations correspond to the encoder outputs and serve as signal-based representations for subsequent semantic conditioning.

**Figure 1:**
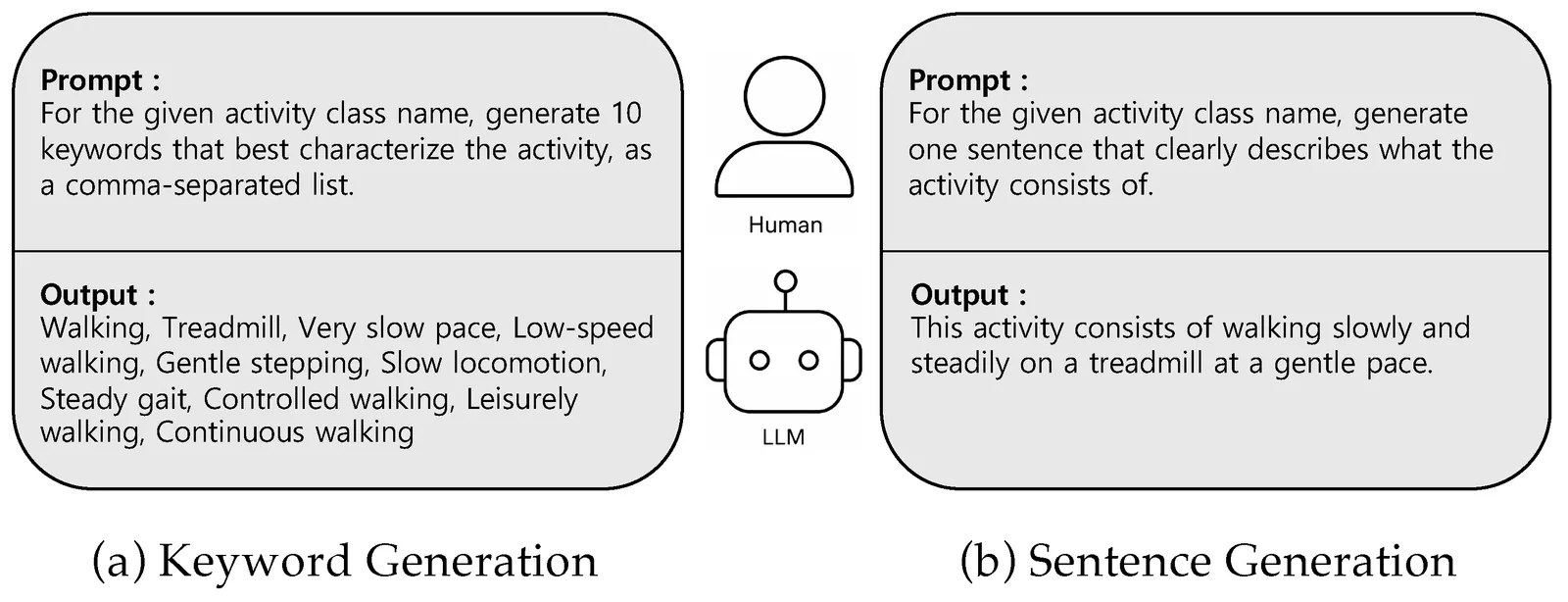
Example of class-wise textual description generation using (**a**) keyword and (**b**) sentence prompt types for “Treadmill 1mph” (Class 0 in the GENEActiv 14-class dataset).

For each activity class c∈{1,…,K}, the corresponding activity label is provided to the LLM as input, and a class-wise textual description $t_{c}$ is generated using the designed prompts, shown in Figure 1. We consider keyword descriptions consisting of terms characterizing the activity and sentence descriptions providing a natural-language description of the activity. A pretrained text encoder $g_{\phi }$ maps each description to a text embedding, $e_{c}=g_{\phi }\left(t_{c}\right)\in R^{d_{t}}$, where $d_{t}$ denotes the dimension of the text embedding. The embeddings of all activity classes are then collected into a text embedding matrix $E=\left[e_{1},e_{2},\ldots ,e_{K}\right]\in R^{K\times d_{t}}$. The text encoder remains frozen throughout training, and the resulting matrix *E* is fixed and used as the common source of semantic information for both teacher- and student-side conditioning.

### 3.2. Semantic Guidance Module

**Figure 2:**
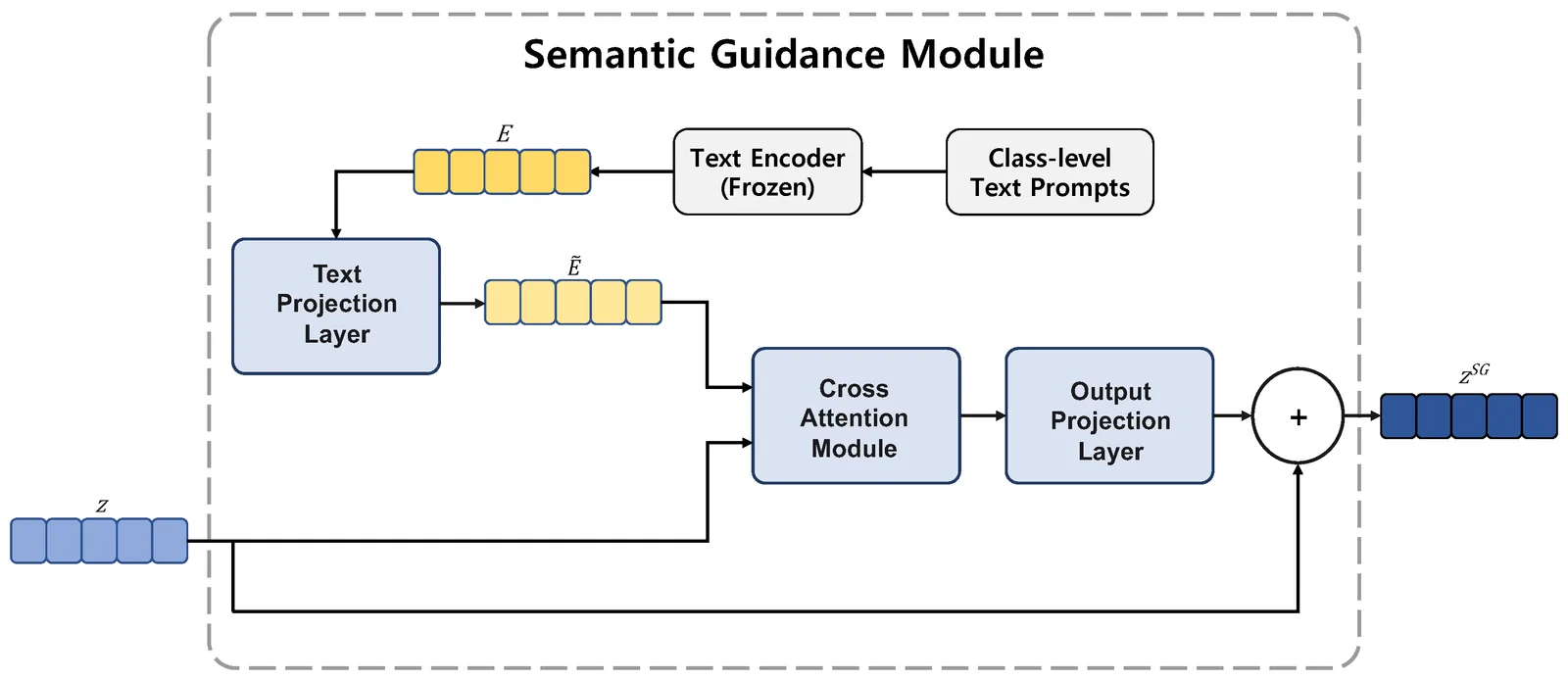
Structure of the SGM used for semantic conditioning of sensor latent representations. The plus sign denotes a residual connection between the original latent representation and the attention output.

We devise a Semantic Guidance Module (SGM) to incorporate language-derived semantics into sensor latent representations. Figure 2 illustrates the overall structure of the SGM, which is used across all semantic conditioning strategies.

Let $z\in R^{d_{z}}$ denote the signal latent representation, which is introduced to the SGM. Here, *z* denotes the latent representation obtained from either the teacher’s or student’s signal, depending on where semantic conditioning is applied. As explained in Section 3.1, the class-wise textual descriptions are first encoded by the frozen text encoder $g_{\phi }$ to construct the text embedding matrix *E*. Next, the matrix is projected into a latent space to match the dimensional size of a signal-based feature through a text projection layer as $\tilde{E}={\mathrm{Proj}}_{text}\left(E\right)\in R^{K\times d_{z}}$. Cross-attention is then performed by treating *z* as the query and $\tilde{E}$ as both the key and value [37], yielding the attentive semantic representation $z^{attn}$ as follows:

(4) $$z^{\mathrm{attn}}=\mathrm{softmax}\left(\frac{q\left(z\right)k{\left(\tilde{E}\right)}^{⊤}}{\sqrt{d_{z}}}\right)v\left(\tilde{E}\right),$$

which is to incorporate textual semantics into the signal-based representation.

Finally, the semantically conditioned representation $z^{\mathrm{SG}}$ is constructed by combining $z^{\mathrm{attn}}$ with the signal-based representation *z* through a residual connection [38] as follows:

(5) $$z^{\mathrm{SG}}=z+{\mathrm{Proj}}_{out}\left(z^{\mathrm{attn}}\right),$$

where ${\mathrm{Proj}}_{out}\left(\cdot \right)$ denotes a learnable linear layer applied to the attention output before the residual connection.

### 3.3. Semantic Conditioning Strategies

**Figure 3:**
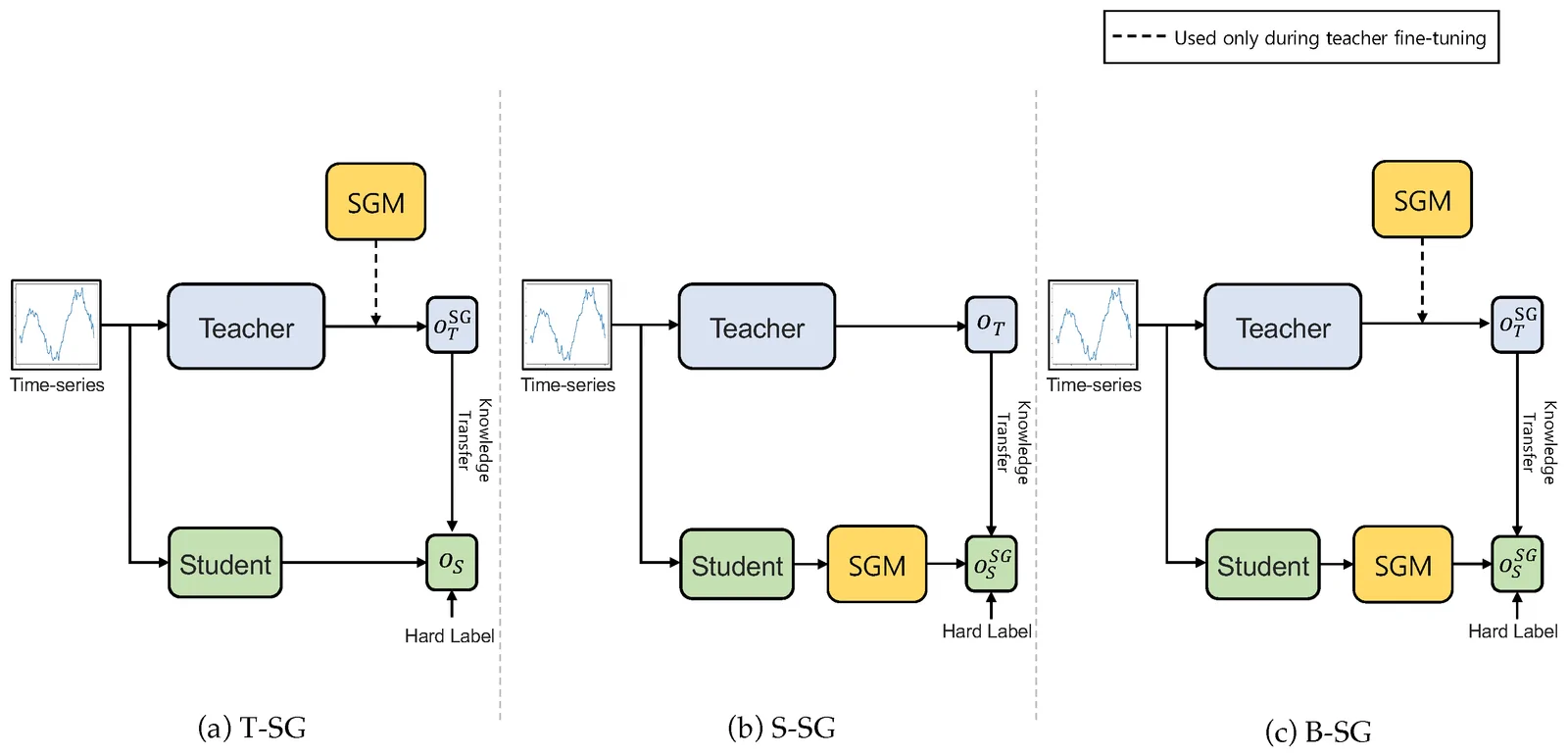
Three semantic conditioning strategies based on KD: (**a**) Teacher-side Semantic Guidance (T-SG), (**b**) Student-side Semantic Guidance (S-SG), and (**c**) Both-side Semantic Guidance (B-SG). The superscript SG indicates logits from a teacher or student trained with the SGM in the corresponding KD strategy.

To examine how semantic information affects KD depending on whether it is introduced to the teacher, the student, or both, we design three semantic conditioning strategies: Teacher-side Semantic Guidance (T-SG), Student-side Semantic Guidance (S-SG), and Both-side Semantic Guidance (B-SG), as illustrated in Figure 3.

**Figure 4:**
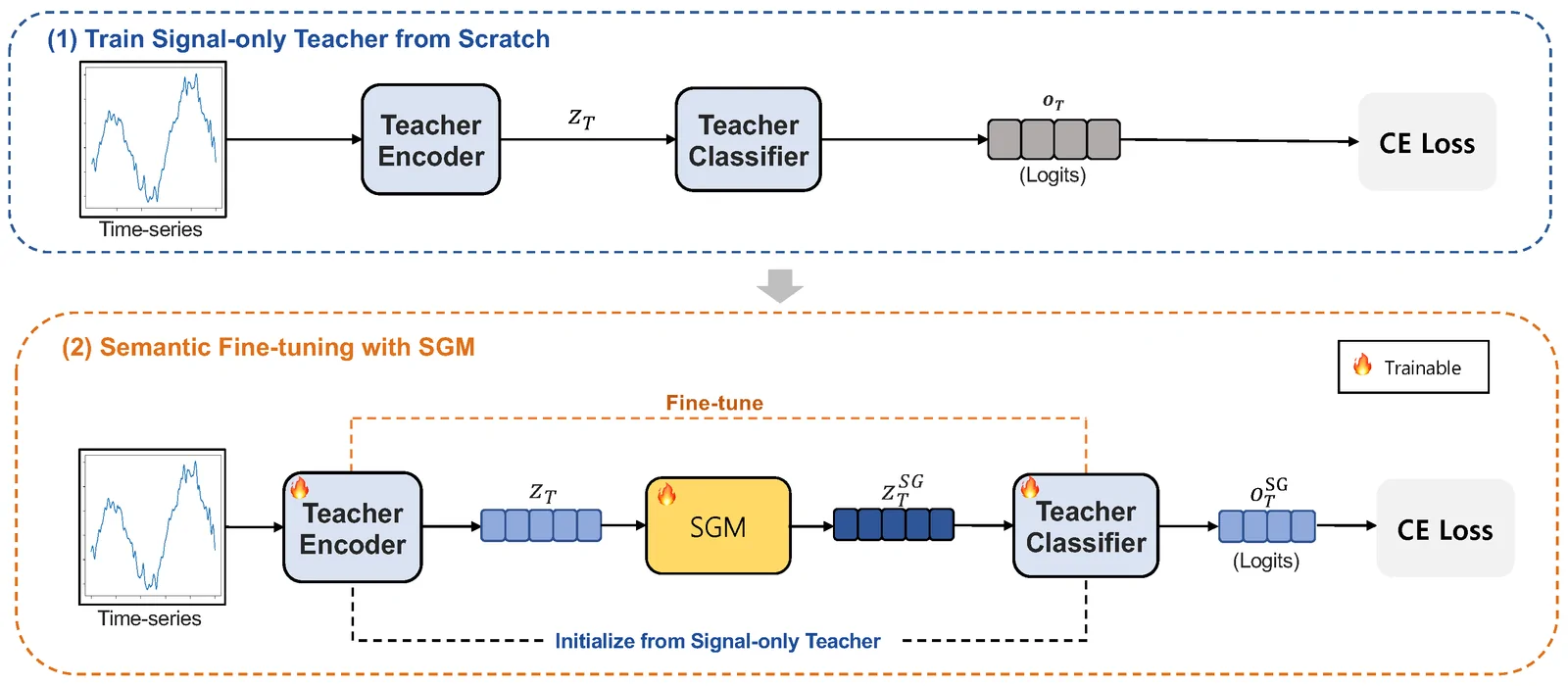
Two-stage procedure for teacher-side semantic conditioning: (**1**) training a signal-only teacher from scratch and (**2**) semantic fine-tuning of the trained teacher with the SGM.

**Teacher-side Semantic Guidance.** T-SG applies semantic conditioning to the teacher through the two-stage training procedure illustrated in Figure 4. First, a teacher is trained from scratch using the time-series alone as input. Second, the proposed SGM is introduced into the pretrained encoder–classifier architecture of the teacher model, and the resulting teacher model is subsequently fine-tuned, with the SGM trained jointly using the standard cross-entropy loss. More specifically, given the teacher latent representation $z_{T}$, the SGM produces the semantically conditioned representation $z_{T}^{\mathrm{SG}}$, which is subsequently passed to the teacher classifier.

During the KD based training phase, the SGM is not utilized, and only the semantically fine-tuned teacher encoder and classifier are retained. Thus, the teacher latent representation $z_{T}$ is directly fed into the classifier to obtain $o_{T}^{\mathrm{SG}}$, which is used for distillation. The student is trained without direct semantic conditioning and produces logits $o_{S}$. Thus, language-derived semantic knowledge is transferred from the teacher to the student, influencing the student indirectly. At inference time, the logits $o_{S}$ of the trained student model are used for prediction.

**Student-side Semantic Guidance.** In S-SG, a teacher trained from scratch using only time-series data is used as the pretrained model during KD, providing knowledge through its logits, $o_{T}$. The SGM is applied to the student latent representation during KD, producing the student logits, $o_{S}^{\mathrm{SG}}$. Thus, semantic information directly influences the student during KD. At inference time, the SGM is removed, and $z_{S}$ of the distilled student is fed into the classifier to obtain the logits $o_{S}^{\mathrm{SG}}$ for prediction.

**Both-side Semantic Guidance.** B-SG combines the teacher- and student-side conditioning pathways. The semantically fine-tuned teacher obtained through T-SG is utilized and $z_{T}$ of the pretrained teacher is fed into the classifier, while the student is also conditioned by the SGM during KD. The logits of the teacher, $o_{T}^{\mathrm{SG}}$, and those of the student, $o_{S}^{\mathrm{SG}}$, are used for knowledge transfer. Accordingly, semantic information is introduced for both the teacher and student. At inference time, the SGM is removed, and $z_{S}$ of the distilled student is directly used in the classifier to yield the logits $o_{S}^{\mathrm{SG}}$ for prediction.

For all three strategies, the corresponding teacher and student logits are used for KD, as defined in Section 2.2.

## 4. Experiments

In this section, we investigate how language-derived semantic information affects KD for wearable sensor-based HAR. We describe the wearable sensor-based datasets and implementation details for our experiments. We then show the experimental results with various evaluations.

### 4.1. Data Description

We use wearable sensor-based datasets including GENEActiv [39] and PAMAP2 [40]. They have different sensor configurations, activity classes, and window lengths, which allow us to evaluate the effects of language-derived semantic information under various experimental settings.

#### 4.1.1. GENEActiv Dataset

**Figure 5:**
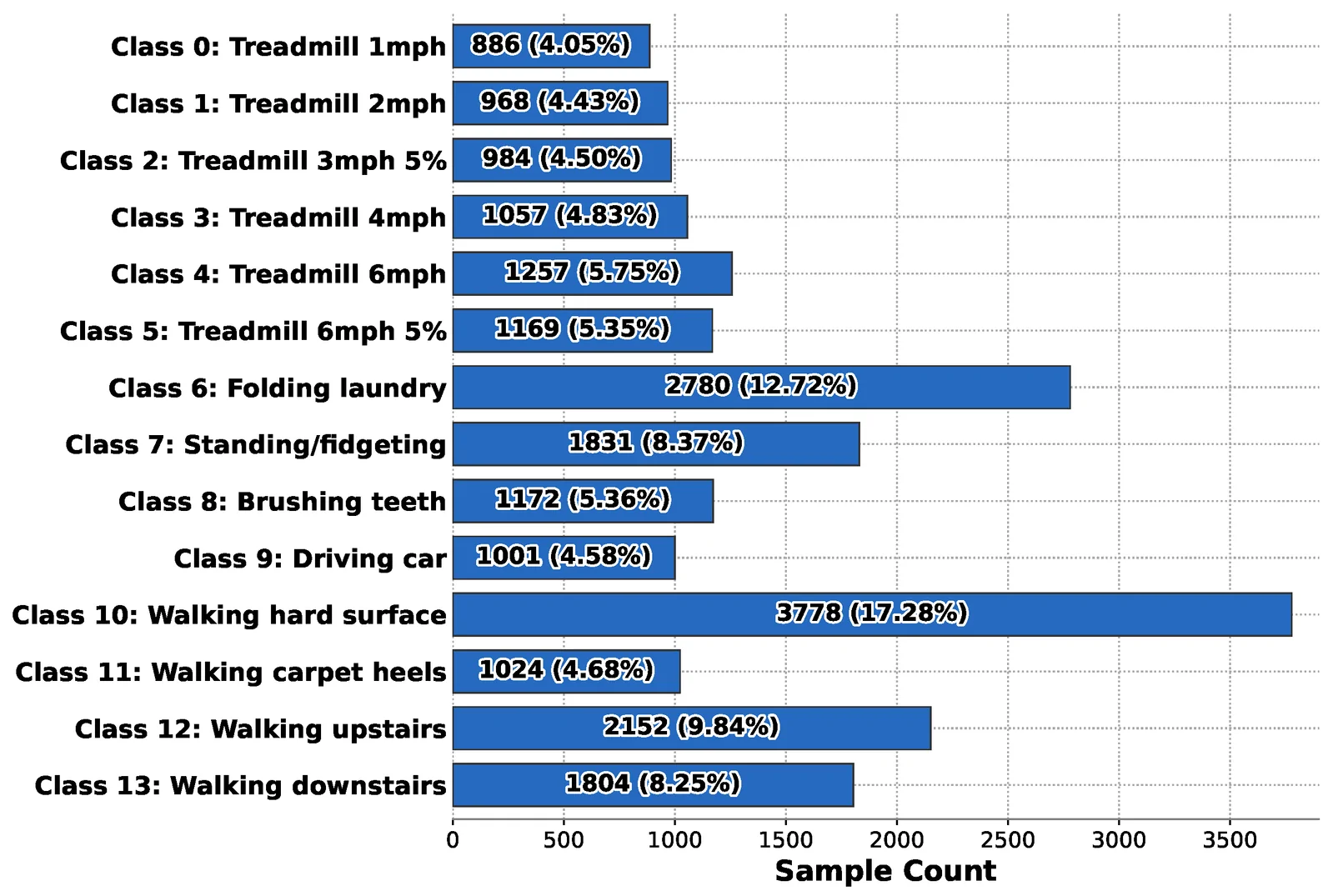
GENEActiv 14-class activity categories and their corresponding per-class sample counts.

GENEActiv [39] consists of tri-axial acceleration signals recorded at 100 Hz using a wrist-mounted wearable sensor. We use 14 activity categories, covering activities such as driving, treadmill walking at different speeds, and daily-life routines. Figure 5 shows the 14 activity categories along with their per-class sample counts. Following the preprocessing protocol used in previous studies [41,42], the continuous recordings are divided into non-overlapping windows of 500 time steps, corresponding to 5 s of sensor data. The training and testing sets are partitioned by 131 and 43 subjects, respectively. After segmentation, the resulting dataset contains approximately 16k training and 6k test samples.

#### 4.1.2. PAMAP2 Dataset

The PAMAP2 dataset [40] contains various wearable sensors collected from 9 subjects. It combines heart-rate and temperature measurements with inertial signals, including accelerometer, gyroscope, and magnetometer measurements, from sensor units positioned on the wrist, chest, and ankle, for a total of 40 sensor channels. In this study, data from 12 daily-activity classes are used. The dataset includes missing sensor readings and class imbalance. The original recordings are downsampled from 100 Hz to 33.3 Hz and divided into windows of 100 time steps with a stride of 22 time steps, corresponding to approximately 3 s per window and a 78% overlap between adjacent segments, following the prior study [43]. For this dataset, we adopt a Leave-One-Subject-Out (LOSO) cross-validation protocol, in which each fold reserves one subject for evaluation while the remaining subjects are used for training. This protocol evaluates generalizability of a model to subjects.

### 4.2. Implementation Details

Both the teacher and student networks were constructed using Wide Residual Network (WRN) architectures [44], which have been widely used as standard backbones in KD studies [42,45]. Their depth and width can be adjusted independently, allowing the effect of teacher capacity on distillation to be examined. We considered four teacher architectures—WRN16-1, WRN16-3, WRN28-1, and WRN28-3—while using WRN16-1 as a student. Since the input data consist of time-series signals, all networks were implemented using 1D convolutions.

For training a model, we used Stochastic Gradient Descent (SGD) with a Nesterov momentum of 0.9, a weight decay of $1\times 10^{-4}$, a batch size of 64, and 200 training epochs. The initial learning rate was set to 0.05, reduced by a factor of 0.2 at epoch 10, and subsequently decayed by a factor of 0.1 every t/3 epochs, where *t* denotes the total number of training epochs. For KD, we used a temperature of τ=4.0 for both datasets and distillation weights of λ=0.7 for GENEActiv and λ=0.99 for PAMAP2, following [42], where these settings were shown to provide stable performance for vanilla KD. Additionally, the teacher fine-tuning stage in T-SG was conducted for 200 epochs.

Textual descriptions were generated using two prompt types, keywords and sentences, with GPT-5.4 [46]. We set 10 keywords as default keyword prompts. The generated descriptions were encoded using the frozen nomic-embed-text-v1.5 model [47] to construct the text embedding matrix *E* described in Section 3.1.

We compared the introduced strategies with diverse prior KD-based methods as baselines, including vanilla KD [16], attention transfer (AT) [48], similarity-preserving distillation (SP) [49], SimKD [50], DIST [51], and the projector-based distillation method [52]. The hyperparameters $\alpha_{\mathrm{AT}}$ and $\gamma_{\mathrm{SP}}$ were set to 1500 and 1000, respectively. Each experiment was conducted three times, and the mean accuracy and standard deviation across the three runs are reported.

We additionally report the model complexity of the four teacher–student pairs on both datasets in terms of FLOPs and parameter counts, as presented in Table 1. Based on these, we compute the compression ratio as the student parameter count divided by the corresponding teacher parameter count to quantify the compactness of the student relative to the teacher. In inference, only the student model is used, as the semantic components are removed after training. Consequently, the semantic conditioning strategies introduce no additional inference-time parameters or computation relative to the corresponding student model.

### 4.3. Analysis of Different Strategies

We conduct experiments with various configurations to comprehensively investigate how language-derived semantic information affects KD and how its effectiveness varies with the location of semantic conditioning, teacher capacity, and data characteristics. We evaluate T-SG, S-SG, and B-SG across different teacher capacities and datasets using both keyword- and sentence-based prompt types.

Table 2 presents the accuracy of student models on the GENEActiv 14-class dataset. Compared with the WRN16-1 student trained from scratch, T-SG consistently improves predictive accuracy across all teacher capacities and prompt types, while also outperforming the prior KD-based methods in most settings. S-SG and B-SG also improve upon the student trained from scratch in most cases, but their improvements are generally smaller and less consistent than those of T-SG. Notably, B-SG does not consistently outperform the single-side strategies (T-SG and S-SG), suggesting that applying semantic conditioning to both the teacher and student does not necessarily provide additional improvements. For T-SG, the teacher accuracies after semantic fine-tuning were 68.89%, 70.02%, 69.53%, and 69.46% for WRN16-1, WRN16-3, WRN28-1, and WRN28-3, respectively, showing only marginal changes from their corresponding scratch-trained accuracies. Despite these small changes in teacher accuracy, distillation results with the semantically fine-tuned teachers (T-SG) resulted in larger improvements in student accuracy. This suggests that T-SG provides semantic-aware knowledge during KD, thereby improving student performance.

The effect of prompt type also varies with the architectural relationship between the teacher and student. Under T-SG, the relatively longer and more descriptive sentence prompts perform better with WRN16-1 and WRN28-1, which have the same width as the WRN16-1 student. In contrast, the shorter and more compact keyword prompts perform better with the wider teachers of WRN16-3 and WRN28-3. Interestingly, this trend suggests that the relative effectiveness of prompt type may depend not simply on teacher capacity, but on architectural differences between the teacher and student, particularly in width. However, this tendency is less pronounced for S-SG and B-SG, suggesting that the effectiveness of different prompt types in relation to teacher–student architecture may depend on where semantic conditioning is applied.

We conduct additional experiments on the GENEActiv 7-class with different window lengths of data to examine the effectiveness of semantic conditioning under different window lengths. Using the same hyperparameters as the 14-class experiments above, we evaluate both 500- and 1000-time-step windows with WRN16-1 and WRN16-3 as teachers.

As shown in Table 3, the effectiveness of the three semantic conditioning strategies differs across window lengths. Under the 500-step window, all three strategies improve predictive accuracy compared with the WRN16-1 student trained from scratch across both teacher settings and prompt types, whereas the improvements from S-SG and B-SG are less consistent under the 1000-step window. Among the three strategies, T-SG shows the most consistent improvements across both window lengths and teacher settings, achieving higher accuracy than S-SG and B-SG in most settings. Interestingly, these improvements were observed despite only marginal changes in teacher accuracy after semantic fine-tuning, similar to the 14-class results. The fine-tuned teacher accuracies were 89.30% and 89.56% for WRN16-1 and WRN16-3 under the 1000-step window and 86.88% and 87.96% under the 500-step window, respectively. The results with T-SG indicate that incorporating semantic information into the teacher can improve student performance during KD.

Under the shorter 500-step window, which provides less temporal context per segment, T-SG shows larger improvements compared with the WRN16-1 student trained from scratch for both teacher settings than under the 1000-step window. T-SG also shows higher student accuracy with WRN16-3 than with WRN16-1 as the teacher under both window lengths. This trend suggests that language-derived semantic information provides a greater benefit when less temporal information is available from the time-series input. The difference between the two teacher settings also indicates that the degree of improvement can depend on teacher capacity.

The results on prompt type also show a relationship with window length. Under T-SG, keyword prompts perform better with both teachers at the 500-step window, whereas sentence prompts perform better at the 1000-step window. This suggests that compact keyword-based semantic cues may be more beneficial with limited temporal information, while the richer descriptions provided by sentence prompts may become more effective with a longer temporal context. Together with the teacher-dependent prompt differences observed in the 14-class setting, these results suggest that prompt effectiveness may depend on both the temporal information available in the time-series input and the architectural relationship between the teacher and student.

Table 4 reports the accuracy of student models on PAMAP2 across four teacher–student combinations. Compared with the student trained from scratch, the semantic conditioning strategies generally improve the student accuracy across the evaluated teacher settings. Among the proposed SG-based methods, although no single strategy consistently achieves the highest accuracy, a common tendency can be observed with respect to the teacher–student gap and prompt type. Sentence prompts generally perform better when the teacher’s architecture is closer to that of the student, whereas keyword prompts become more competitive as the architectural gap between the teacher and student increases. Specifically, sentence prompts achieve the highest accuracy with the WRN16-1, WRN16-3, and WRN28-1 teachers, while T-SG with keyword prompts achieves the highest accuracy with WRN28-3, which differs from the student in both depth and width. Furthermore, consistent with the GENEActiv results, T-SG improved student accuracy despite only marginal changes in teacher accuracy after semantic fine-tuning. The fine-tuned teacher accuracies were 85.32%, 85.85%, 84.87%, and 84.47% for WRN16-1, WRN16-3, WRN28-1, and WRN28-3, respectively, while their distilled students achieved larger accuracy gains.

Under T-SG, sentence prompts perform better with WRN16-1 and WRN28-1 teachers, which have the same width as the WRN16-1 student, whereas keyword prompts perform better with the wider WRN16-3 and WRN28-3 teachers. This tendency is consistent with the results observed on GENEActiv, where T-SG with keyword prompts tends to outperform the other strategies when the capacity gap between the teacher and student is large.

Despite this common tendency, the PAMAP2 and GENEActiv results differ in several cases that may be caused by the characteristics of the data. PAMAP2 uses a substantially shorter window length and includes multiple sensor modalities with a larger number of input channels than GENEActiv. Consequently, each PAMAP2 segment contains less temporal context while providing signal information from a larger set of input channels. In particular, the combination of limited temporal context, richer multi-channel signal information, and subject-independent evaluation may change how complementary language-derived semantic information contributes to the signal-based representations and the subsequent KD process. Furthermore, PAMAP2 is evaluated using a leave-one-subject-out protocol, requiring the model to generalize to an unseen subject in each fold. Therefore, the effectiveness of semantic conditioning appears to depend not only on the teacher–student architecture and prompt type, but also on the characteristics of the input signals and the evaluation setting.

**Figure 6:**
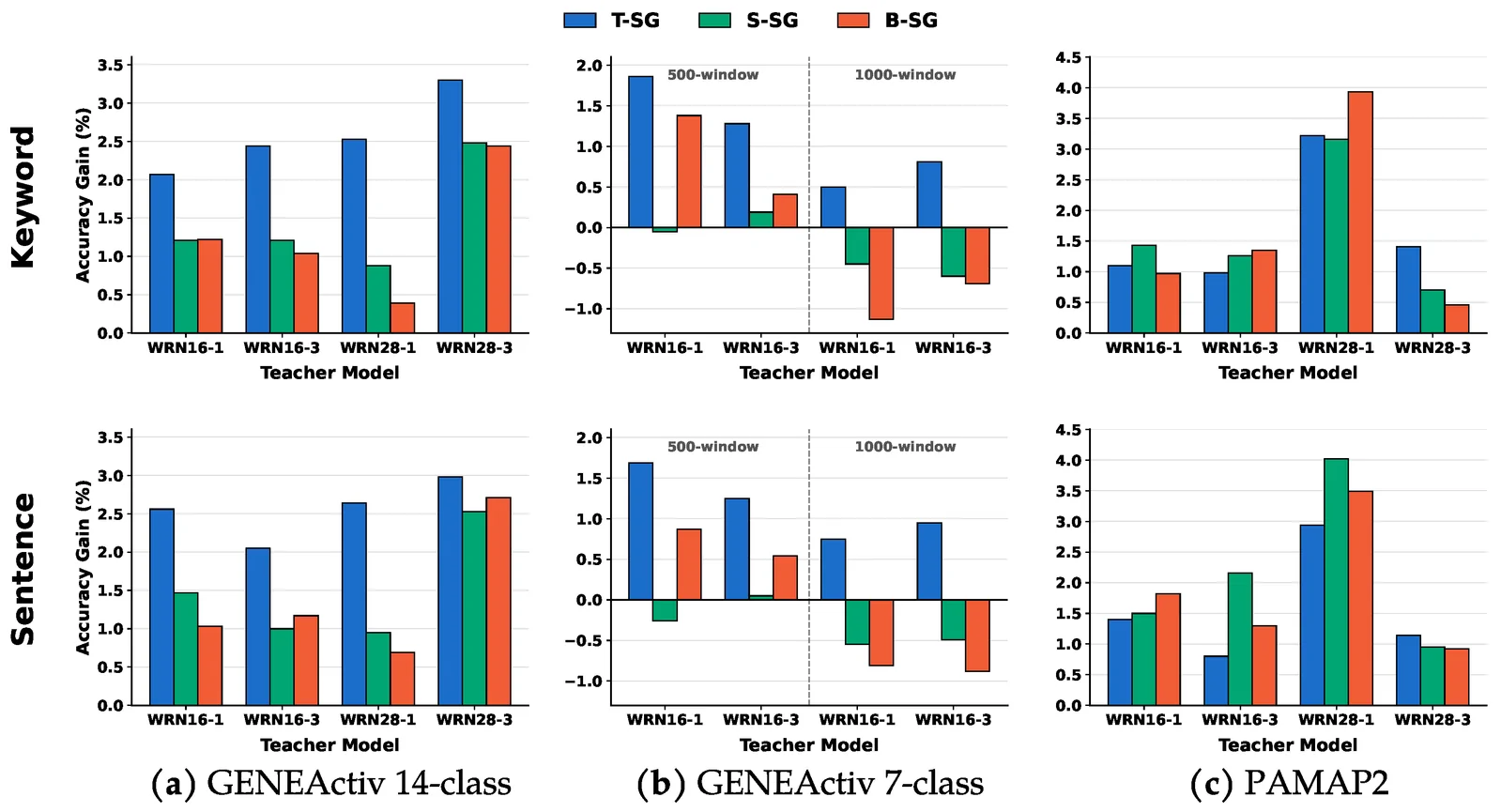
Accuracy gains over vanilla KD across the three semantic conditioning strategies using keyword and sentence prompts: (**a**) GENEActiv 14-class, (**b**) GENEActiv 7-class, and (**c**) PAMAP2.

To further investigate the effects of semantic guidance, Figure 6 presents the accuracy gains over vanilla KD across the GENEActiv 14-class, GENEActiv 7-class, and PAMAP2.

For GENEActiv 14-class, all three semantic conditioning strategies perform better than vanilla KD across all teacher models for both keyword and sentence prompts. T-SG generally shows larger gains, with the gains tending to become larger for teacher architectures that differ more from the student. This trend is observed for both prompt types, indicating that the advantage of teacher-side semantic conditioning is not limited to a specific form of semantic description.

A similar advantage of T-SG is observed on the GENEActiv 7-class dataset, where it consistently outperforms vanilla KD across both window lengths, teacher models, and prompt types. In contrast, S-SG and B-SG do not consistently provide additional gains. In particular, with the 1000-step window, both strategies show negative gains for both teacher models under keyword and sentence prompts, whereas T-SG maintains positive gains. This contrast is less pronounced with the shorter 500-step window. This suggests that the effectiveness of each conditioning strategy may depend on the amount of temporal information contained in the input.

On the other hand, a different tendency is presented on PAMAP2. All three strategies generally provide positive gains over vanilla KD, with the largest improvements observed with WRN28-1 for both prompt types. In contrast to the trend observed on the other datasets, T-SG does not achieve the largest gain on PAMAP2. T-SG yields the highest improvement among the three strategies only with WRN28-3, whereas S-SG or B-SG provides larger gains with the other teacher models.

Overall, although the degree of improvement differs between keyword and sentence prompts, T-SG consistently improves performance in most cases and tends to be particularly effective when the teacher and student differ in depth, with these major trends broadly maintained.

### 4.4. Analysis of Performance Across Activities

To further investigate how semantic conditioning affects individual activities beyond overall classification accuracy, we analyze both activity-level recognition performance and latent feature distributions. Specifically, we examine class-wise F1-scores to evaluate the recognition performance of individual activities and analyze the student latent feature space using t-SNE [53] and the V-Score [54].

#### 4.4.1. F1-Score Analysis

Since the F1-score jointly reflects precision and recall, we use class-wise F1-scores to examine how the effects of semantic conditioning vary across individual activities.

**Figure 7:**
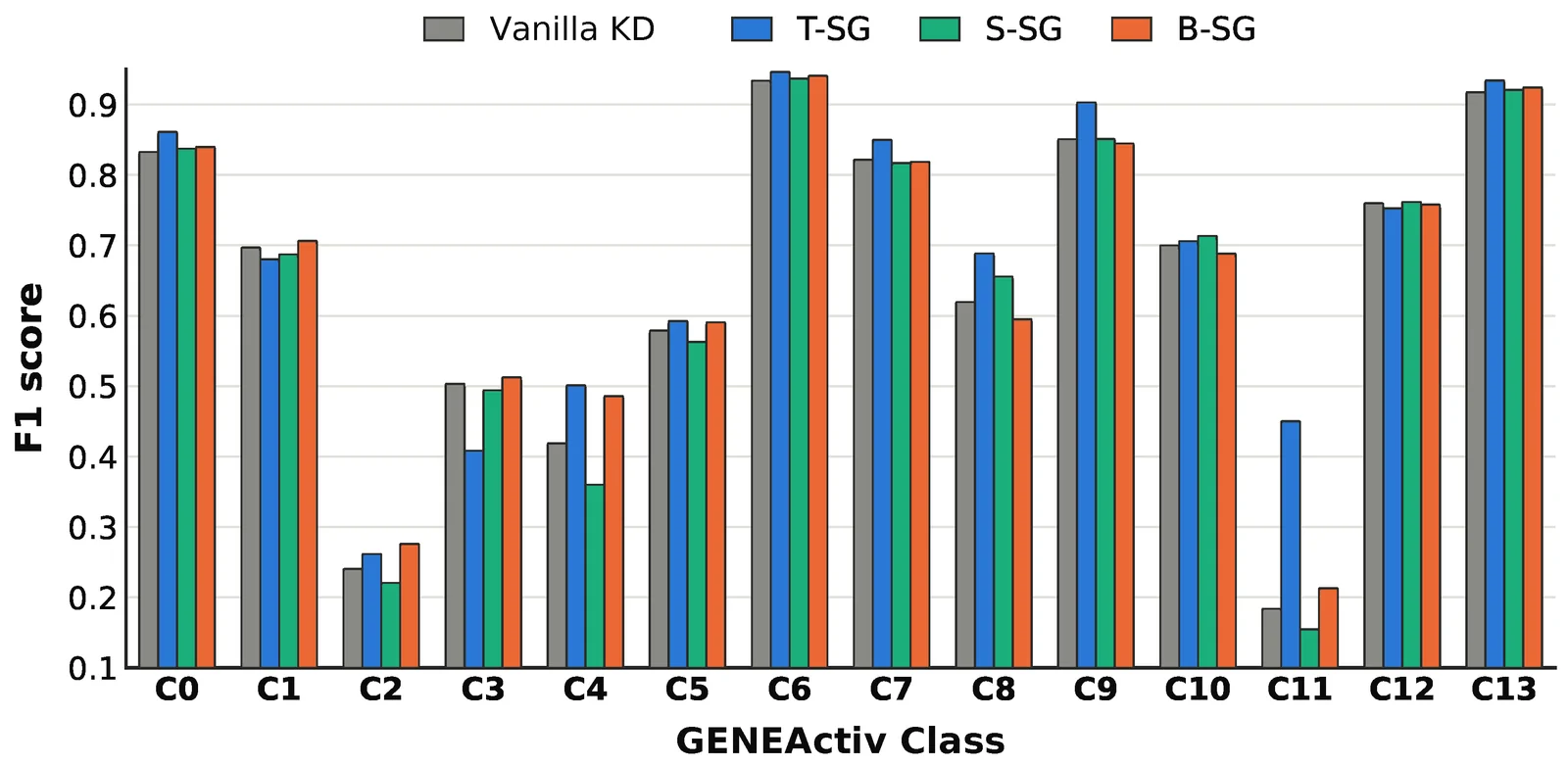
Per-class F1 scores on GENEActiv for vanilla KD and the three semantic conditioning strategies under the WRN16-3 teacher using keyword prompts. Class indices follow the activity ordering shown in Figure 5. Across the entire testing set, the corresponding weighted F1-scores are 0.6667, 0.7225, 0.7036, and 0.7029 for vanilla KD, T-SG, S-SG, and B-SG, respectively.

As shown in Figure 7, T-SG achieves the highest F1-scores for most activity classes, although the degree of improvement varies across activities. The most pronounced improvement is observed for Class 11 (Walking on carpet in heels). Although vanilla KD performed better for some classes, the use of semantic guidance achieved better overall performance and greater total gains.

Across the entire testing set, all three semantic conditioning strategies achieve higher weighted F1-scores than vanilla KD, with T-SG achieving the highest value. This suggests that semantic guidance can better capture and transfer class-relevant knowledge from the teacher to the student, leading to more effective knowledge distillation overall.

#### 4.4.2. t-SNE Analysis

**Figure 8:**
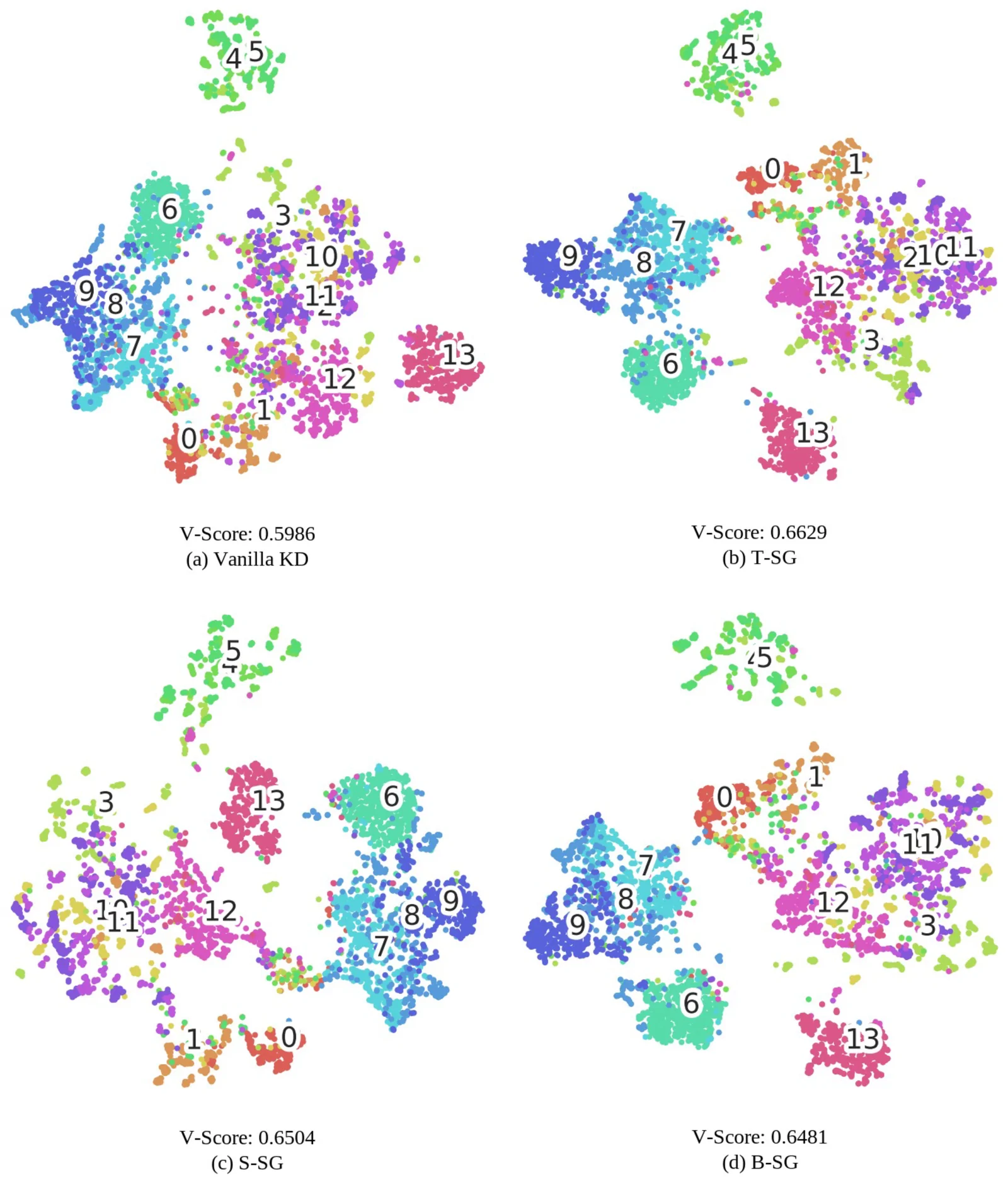
t-SNE visualizations of the student latent feature distributions on GENEActiv under the WRN16-3 teacher using keyword prompts: (**a**) vanilla KD, (**b**) T-SG, (**c**) S-SG, and (**d**) B-SG. Different colors represent the 14 activity classes, and the numbers (0–13) indicate their corresponding class indices. The V-Score is reported for each strategy.

Figure 8 visualizes the t-SNE plots [53] to compare the student latent feature distributions under vanilla KD and the three semantic conditioning strategies. The accompanying V-Score [54] provides a quantitative measure of clustering quality by jointly considering cluster homogeneity and completeness.

As shown in Figure 8, semantic conditioning improves the separation between activity classes compared with vanilla KD. As reported with V-Score, the distilled model with semantic guidance achieves better clustering quality. In detail, T-SG achieves the highest score, 0.6629.

Under vanilla KD, Class 7 (Standing/fidgeting), Class 8 (Brushing teeth), and Class 9 (Driving Car) occupy highly overlapping regions. For the three semantic conditioning strategies, these classes become more distinguishable than vanilla KD. Additionally, Class 12 (Walking upstairs) also tends to form a more compact cluster under T-SG than under vanilla KD.

However, Class 4 (Treadmill 6 mph) and Class 5 (Treadmill 6 mph, 5% incline), which correspond to treadmill activities performed at the same speed but with different inclines, remain substantially overlapped across the strategies. A similar tendency is observed for Class 10 (Walking on a hard surface) and Class 11 (Walking on carpet in heels), which share the same underlying activity semantics of walking but differ in contextual attributes such as the ground surface. Semantic information that captures class-specific distinctions may be more effective in enhancing separation between classes.

### 4.5. Analysis of Model Reliability

To further examine the effects of semantic conditioning beyond predictive accuracy, we analyze model reliability. To assess model reliability in terms of prediction confidence, we use the Expected Calibration Error (ECE) [55], which measures the discrepancy between predicted confidence and empirical accuracy, with lower values indicating better reliability.

As shown in Table 5, all three semantic conditioning strategies achieve lower ECE than vanilla KD across both teacher–student combinations and prompt types, indicating that semantic conditioning enables KD to improve student reliability in addition to predictive performance. Among the three semantic guidance-based strategies, S-SG consistently achieves the lowest ECE, whereas T-SG generally provides higher predictive accuracy. This result shows that higher predictive accuracy does not necessarily correspond to lower ECE.

Previous studies have similarly shown that predictive accuracy and calibration characterize different aspects of model performance and do not necessarily improve together [55,56]. A model can correctly classify more samples while still producing confidence estimates that are less aligned with empirical correctness. This provides a possible explanation for the difference between T-SG and S-SG: T-SG achieves higher predictive accuracy, while S-SG exhibits higher reliability.

A consistent tendency is observed with respect to prompt type. Keyword prompts yield lower ECE than sentence prompts across both teacher capacities and all three semantic conditioning strategies, although neither prompt type shows a consistent advantage in predictive accuracy. Keyword prompts provide semantic cues in a compact form that emphasizes class-specific attributes. In contrast, sentence prompts contain richer linguistic descriptions that encode multiple semantic attributes and their contextual relationships [57,58,59]. However, richer semantic descriptions do not necessarily provide more discriminative information, as overlapping semantic cues may introduce ambiguity among semantically similar classes [57]. This may partly explain why the additional linguistic information provided by sentence prompts does not consistently improve accuracy and is associated with higher ECE in our experiments. These results suggest that the form of semantic information may affect predictive performance and model reliability differently. That is, the additional linguistic information provided by sentence prompts does not necessarily result in better reliability.

### 4.6. Analysis of Semantic Density

Focusing on T-SG, we examine how the number of keywords and the distillation temperature affect semantic-guided KD. The number of keywords is used as an operational measure of semantic density, reflecting the amount of class-level textual information provided to the model, while the temperature τ controls the smoothness of the knowledge transferred from the teacher.

**Figure 9:**
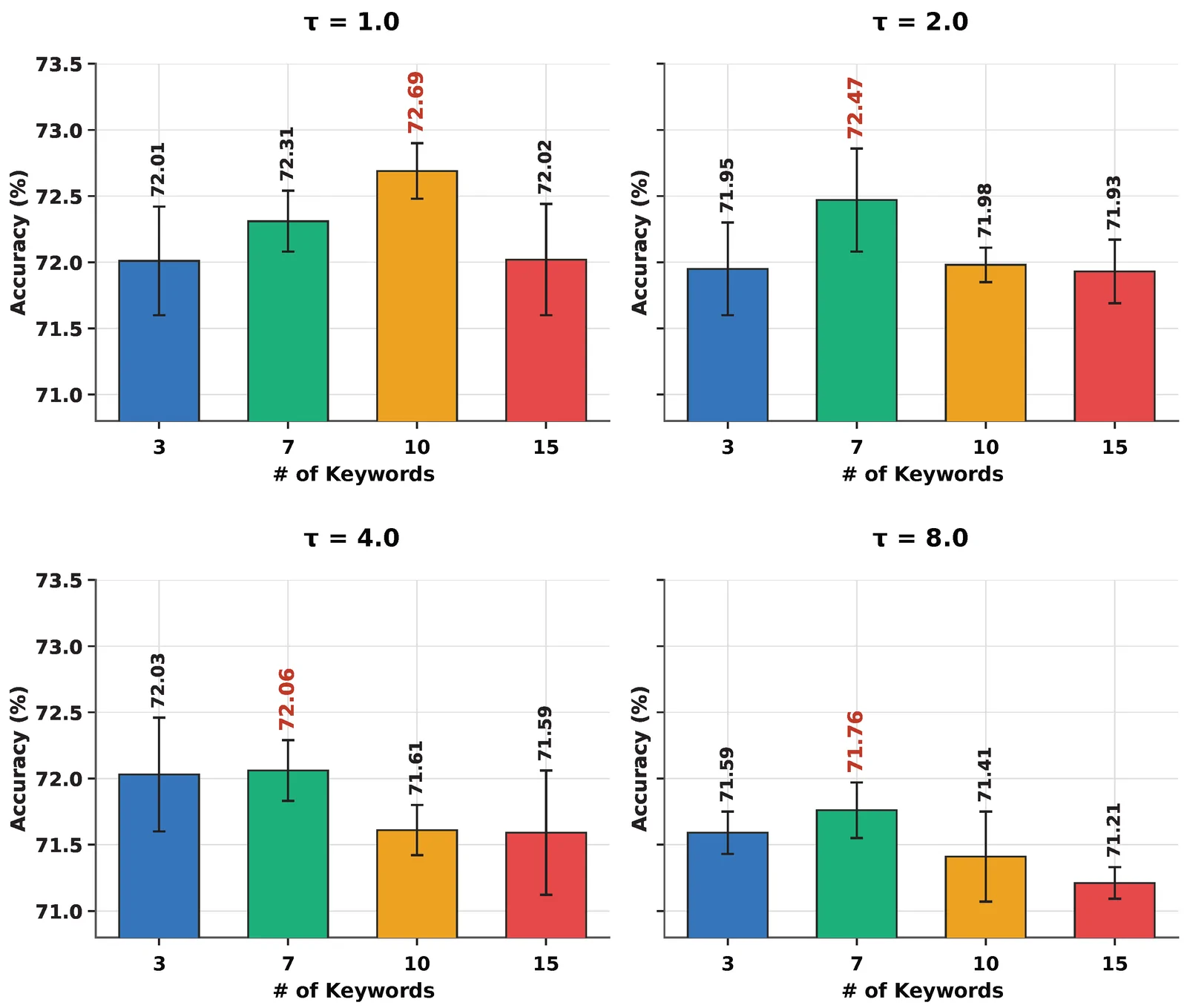
Accuracy on GENEActiv under different numbers of keywords and distillation temperatures τ for T-SG with activity-based keyword prompts. Results show the average accuracy across four teacher–student combinations. The values highlighted in red denote the highest average accuracy at each distillation temperature.

Figure 9 presents the averaged accuracy of T-SG on GENEActiv across four teacher–student combinations. The results show that increasing the number of keywords does not consistently improve predictive accuracy across the evaluated distillation temperatures, suggesting that higher semantic density does not necessarily lead to better performance.

In T-SG, incorporating semantic information appears to increase the smoothness of the signal-based representations, which explains why a lower distillation temperature is more effective. This phenomenon is consistent with previous studies showing that introducing additional information into the latent space can improve KD generalizability by modifying the smoothness of the learned representations, particularly when using a lower distillation temperature [60]. Therefore, these findings suggest that effective KD can be achieved by incorporating an appropriate amount of textual semantic information while avoiding excessive smoothing through the distillation temperature.

### 4.7. Processing Time

We compare the inference time of various models on the GENEActiv testing set, consisting of approximately 6k samples. The inference was performed with a batch size of 1. All experiments were conducted on a desktop equipped with an AMD Ryzen Threadripper PRO 5955WX (16 cores/32 threads, base clock 3.6 GHz), 256 GB of RAM, and an NVIDIA GeForce RTX 4090 GPU with 24 GB of memory. We use WRN16-1 student models distilled from WRN16-3 teacher models for KD methods. As shown in Table 6, student model requires less inference time on both GPU and CPU than the WRN28-3 model learned from scratch. All three distilled student from the proposed semantic-guided KD strategies also achieve higher accuracy than both the WRN28-3 model learned from scratch and the distilled student from vanilla KD.

Interestingly, the student model requires less processing time on the CPU (9.05 s) than on the GPU (10.94 s). This may be attributed to the small model size and the batch size of 1, which limit GPU parallelism while making memory transfer and GPU operation overhead relatively more significant [61,62].

## 5. Discussion

The architectural relationship between the teacher and student provides an important perspective for interpreting the effects of semantic conditioning in KD. Previous studies have shown that larger or more accurate teachers do not necessarily produce better students, indicating that KD performance cannot be explained by teacher capacity alone [45]. In our experiments, a common tendency is observed between the teacher–student architectural gap and the effectiveness of different prompt types across GENEActiv and PAMAP2. Sentence prompts generally perform better when the teacher architecture is closer to that of the student, whereas keyword prompts become more competitive as the architectural gap increases. This finding extends the importance of the teacher–student relationship to semantic conditioning, indicating that the effectiveness of language-derived semantic information may depend not only on the prompt type but also on the architectural conditions under which semantic knowledge is transferred during KD.

The effectiveness of semantic information is also related to the information available from the input signals. In the GENEActiv 7-class experiments, keyword prompts perform better under the shorter temporal window, whereas sentence prompts perform better under the longer temporal window. This tendency suggests that compact semantic cues may be more beneficial when temporal information from the input signal is limited, while richer semantic descriptions may become more effective when longer temporal context is available. The degree of performance improvement also varies across activity classes. In particular, the activity-level analysis shows that semantic conditioning improves the distinction of several activities while substantial overlap remains between activities sharing similar motion characteristics. Taken together, these results suggest that the contribution of semantic information may depend on the extent to which it provides complementary information beyond that already available from the input signals. This observation is consistent with human information processing, where sensory evidence is interpreted together with prior semantic knowledge rather than in isolation [11,12]. Similarly, our results suggest that language-derived semantics can provide complementary information to sensor-based representations, with their contribution varying according to the information available from the input signals.

The additional analyses indicate that semantic conditioning can affect different aspects of the student model in different ways. T-SG achieves the highest V-Score and generally provides the strongest predictive accuracy on GENEActiv, whereas S-SG achieves better model reliability. This difference suggests that improving classification performance does not necessarily lead to the greatest improvement in prediction reliability.

The analysis of semantic density further indicates a possible interaction between semantic conditioning and the smoothness involved in KD. Increasing the number of keywords may affect the smoothness of the signal-based representations by providing a greater amount of semantic information. Previous work [60] has shown that excessive smoothing can hinder effective knowledge transfer and that the degree of smoothness needs to be appropriately balanced with that controlled by the distillation temperature. Similarly, our results show that increasing semantic density does not continuously improve performance and that its preferred level varies with distillation temperature. This finding indicates that effective semantic conditioning for signal analysis in KD requires an appropriate balance in the amount of semantic information provided, as excessive semantic information may introduce an undesired smoothing effect. Thus, simply increasing the amount of semantic information does not necessarily lead to better performance.

In this paper, to enable controlled comparisons across different semantic conditioning configurations, including the location of semantic guidance, prompt type, and semantic density, the same LLM-generated textual information was fixed across all experiments. However, textual information generated by LLMs can vary across independent generations, and such generation-level variability was not examined in this study. Therefore, the observed effects of semantic conditioning are limited to the fixed textual information considered in our experiments, and their robustness to independently generated textual information remains to be further investigated.

## 6. Conclusions

In this study, we investigated the role of language-derived semantics in KD for wearable sensor data analysis. We devised three different strategies for leveraging semantic guidance during distillation. Our experiments demonstrated that incorporating language-derived semantics into the distillation process can provide complementary knowledge that improves KD performance. In particular, in most cases, when semantic conditioning is applied to the teacher, the resulting distilled student achieves better performance than those obtained using the other methods and strategies. We further explored the effectiveness of semantic guidance across different prompt types and examined its relationship with the temporal information provided by the time-series input. When time-series input provides sufficient temporal information, sentence-based semantic representations enable more effective guidance for improving distillation, resulting in a better-performing student model. Also, we found that an appropriate level of semantic density is important for effective distillation.

Conclusively, we found that the effectiveness of language-derived semantics in KD is determined not only by the presence or amount of semantic information but also by how semantic knowledge interacts with the teacher–student configuration and the information available from the time-series input. By combining sensor-derived information with prior semantic knowledge, the proposed approach reflects a general principle of human information processing, in which sensory evidence is interpreted in conjunction with existing knowledge. These findings provide a broader understanding of how language-derived semantic knowledge can be incorporated into KD to generate a lightweight model for wearable sensor analysis.

In future work, we will extend the current investigation by considering more diverse forms of language-derived semantic information beyond class-level keyword and sentence representations. As part of this extension, the robustness of semantic-guided KD will be examined across multiple independently generated textual descriptions to account for the variability of LLM-generated semantic information. This broader investigation will also consider different time-series tasks to better understand how the contribution of semantic information varies across application settings.

## Figures and Tables

**Table 1:** Description of the teacher and student networks used in this study. Compression ratio is computed as the student’s value divided by the teacher’s. WideResNet is denoted as WRN(depth)-(width).

DB	Teacher	Student	FLOPs		# of Params		Compression
			Teacher	Student	Teacher	Student	Ratio
GENEActiv	WRN16-1	WRN16-1	11.03 M	11.03 M	0.06 M	0.06 M	–
	WRN16-3		93.95 M		0.54 M		11.11%
	WRN28-1		22.22 M		0.13 M		46.15%
	WRN28-3		192.01 M		1.12 M		5.36%
PAMAP2	WRN16-1	WRN16-1	2.39 M	2.39 M	0.06 M	0.06 M	–
	WRN16-3		19.00 M		0.54 M		11.11%
	WRN28-1		4.64 M		0.13 M		46.15%
	WRN28-3		38.64 M		1.12 M		5.36%

**Table 2:** Accuracy (%) on GENEActiv 14-class across teacher capacities. Values in parentheses indicate the accuracy of the corresponding models trained from scratch. Best result in each column is in **bold**.

Teacher		WRN16-1	WRN16-3	WRN28-1	WRN28-3
		(68.87)	(69.99)	(69.52)	(69.42)
Student		WRN16-1 (68.87 ± 0.49)			
KD	Vanilla KD	69.71 ± 0.38	69.50 ± 0.10	68.32 ± 0.63	68.58 ± 0.66
	AT	68.21 ± 0.64	69.79 ± 0.36	68.09 ± 0.24	67.73 ± 0.27
	SP	67.20 ± 0.36	67.85 ± 0.24	68.71 ± 0.46	67.39 ± 0.49
	SimKD	69.39 ± 0.18	69.89 ± 0.11	68.92 ± 0.40	68.80 ± 0.38
	DIST	68.20 ± 0.28	69.71 ± 0.15	69.23 ± 0.19	68.18 ± 0.60
	Projector	69.64 ± 0.53	70.28 ± 0.38	69.43 ± 0.47	69.09 ± 0.38
Keyword	T-SG	71.78 ± 0.32	**71.94** ± 0.38	70.85 ± 0.13	**71.88** ± 0.24
	S-SG	70.92 ± 0.21	70.71 ± 0.16	69.20 ± 0.34	71.06 ± 0.50
	B-SG	70.93 ± 0.55	70.54 ± 0.67	68.71 ± 0.87	71.02 ± 0.31
Sentence	T-SG	**72.27** ± 0.45	71.55 ± 0.42	**70.96** ± 0.30	71.56 ± 0.78
	S-SG	71.18 ± 0.04	70.50 ± 0.20	69.27 ± 0.33	71.11 ± 0.47
	B-SG	70.74 ± 0.34	70.67 ± 0.50	69.01 ± 0.41	71.29 ± 0.95

**Table 3:** Accuracy (%) on GENEActiv 7-class across window sizes and teacher capacities. Values in parentheses indicate the accuracy of the corresponding models trained from scratch. Best result in each column is in **bold**.

Window		1000		500	
Teacher		WRN16-1	WRN16-3	WRN16-1	WRN16-3
		(89.29 ± 0.32)	(89.53 ± 0.15)	(86.83 ± 0.15)	(87.95 ± 0.25)
Student		WRN16-1 (89.29 ± 0.32)		WRN16-1 (86.83 ± 0.15)	
KD	Vanilla KD	89.79 ± 0.32	89.88 ± 0.07	87.44 ± 0.53	88.16 ± 0.15
	AT	90.10 ± 0.49	90.32 ± 0.09	87.25 ± 0.22	87.60 ± 0.22
	SP	87.08 ± 0.56	88.47 ± 0.19	87.65 ± 0.11	87.69 ± 0.18
	SimKD	90.25 ± 0.22	90.47 ± 0.32	87.24 ± 0.09	88.16 ± 0.37
	DIST	90.18 ± 0.31	90.20 ± 0.39	87.62 ± 0.02	87.05 ± 0.31
	Projector	90.01 ± 0.70	90.23 ± 0.36	87.75 ± 0.90	87.93 ± 0.72
Keyword	T-SG	90.29 ± 0.55	90.69 ± 0.51	**89.30** ± 0.36	**89.44** ± 0.24
	S-SG	89.34 ± 0.29	89.28 ± 0.46	87.43 ± 0.49	88.35 ± 0.20
	B-SG	88.66 ± 0.25	89.19 ± 1.20	88.82 ± 0.37	88.57 ± 0.11
Sentence	T-SG	**90.54** ± 0.36	**90.83** ± 0.19	89.13 ± 0.46	89.41 ± 0.11
	S-SG	89.24 ± 0.33	89.39 ± 0.47	87.18 ± 0.67	88.19 ± 0.20
	B-SG	88.98 ± 0.54	89.00 ± 1.32	88.31 ± 0.42	88.70 ± 0.18

**Table 4:** Accuracy (%) on PAMAP2 across teacher capacities. Values in parentheses indicate the accuracy of the corresponding models trained from scratch. Best result in each column is in **bold**.

Teacher		WRN16-1	WRN16-3	WRN28-1	WRN28-3
		(85.27)	(85.80)	(84.81)	(84.46)
Student		WRN16-1 (82.99 ± 2.50)			
KD	Vanilla KD	85.96 ± 2.19	86.50 ± 2.21	84.92 ± 2.45	86.26 ± 2.40
	Projector	86.61 ± 2.04	85.07 ± 2.32	84.59 ± 2.53	86.78 ± 2.26
Keyword	T-SG	87.06 ± 2.60	87.48 ± 2.57	88.14 ± 2.53	**87.67** ± 2.51
	S-SG	87.39 ± 2.37	87.76 ± 2.56	88.08 ± 2.55	86.96 ± 2.78
	B-SG	86.93 ± 2.44	87.85 ± 2.61	88.85 ± 2.27	86.72 ± 2.72
Sentence	T-SG	87.36 ± 2.62	87.30 ± 2.39	87.86 ± 2.52	87.40 ± 2.53
	S-SG	87.46 ± 2.41	**88.66** ± 2.33	**88.94** ± 2.33	87.21 ± 2.75
	B-SG	**87.78** ± 2.16	87.80 ± 2.39	88.41 ± 2.54	87.18 ± 2.67

**Table 5:** Expected Calibration Error (ECE, %; lower is better) on GENEActiv using WRN16-3 and WRN28-1 teachers with all methods distilling into a WRN16-1 student. Lowest (best) ECE in each column is in **bold**.

Teacher		WRN16-3	WRN28-1
Vanilla KD		3.312	3.069
SG (Keyword)	T-SG	2.826	2.758
	S-SG	**2.624**	**2.598**
	B-SG	2.795	2.751
SG (Sentence)	T-SG	2.886	2.789
	S-SG	2.703	2.674
	B-SG	2.801	2.756

**Table 6:** Classification accuracy and processing time of various models on GENEActiv. WRN16-3 and WRN16-1 are used as the teacher and student models for all KD methods, and keyword prompts are used for the proposed semantic-guided methods. LS denotes learning from scratch. Bold indicates the highest accuracy and the shortest processing times.

Model	LS	KD			
	WRN28-3	Vanilla	T-SG	S-SG	B-SG
Accuracy (%)	69.42	69.50	**71.83**	70.71	70.53
GPU (s)	19.29	**10.94**			
CPU (s)	28.36	**9.05**			

## Data Availability

GENEActiv data was collected by the Exercise Science and Health Promotion Department of Arizona State University and is not currently approved for public release. PAMAP2 is a public dataset available at https://archive.ics.uci.edu/dataset/231/pamap2+physical+activity+monitoring (accessed on 1 September 2026).

## References

[1] Patel S., Park H., Bonato P., Chan L., Rodgers M. (2012). A review of wearable sensors and systems with application in rehabilitation. J. Neuroeng. Rehabil..

[2] Perumal T., Ramanujam E., Suman S., Sharma A., Singhal H. (2023). Internet of Things Centric-Based Multiactivity Recognition in Smart Home Environment. IEEE Internet Things J..

[3] Gu T., Tang M. (2025). Indoor Abnormal Behavior Detection for the Elderly: A Review. Sensors.

[4] Zhang S., Li Y., Zhang S., Shahabi F., Xia S., Deng Y., Alshurafa N. (2022). Deep learning in human activity recognition with wearable sensors: A review on advances. Sensors.

[5] Wei H., Hua J., Jiang Y., Zhu W., Cheng W., Shi Y., Pan L. (2026). Seamless Human–Computer Interaction Enabled by Wearable Biointerfaces and Intelligent Systems. Biomimetics.

[6] Gupta S. (2021). Deep learning based human activity recognition (HAR) using wearable sensor data. Int. J. Inf. Manag. Data Insights.

[7] Bulling A., Blanke U., Schiele B. (2014). A Tutorial on Human Activity Recognition Using Body-Worn Inertial Sensors. ACM Comput. Surv..

[8] Wang J., Chen Y., Hao S., Peng X., Hu L. (2019). Deep learning for sensor-based activity recognition: A survey. Pattern Recognit. Lett..

[9] Kunze K., Lukowicz P. (2014). Sensor Placement Variations in Wearable Activity Recognition. IEEE Pervasive Comput..

[10] Hurtado-Perez A.E., Toledano-Ayala M., Cruz-Albarran I.A., Lopez-Zúñiga A., Moreno-Perez J.A., Álvarez-López A., Rodriguez-Resendiz J., Perez-Ramirez C.A. (2025). Use of Technologies for the Acquisition and Processing Strategies for Motion Data Analysis. Biomimetics.

[11] Summerfield C., de Lange F.P. (2014). Expectation in perceptual decision making: Neural and computational mechanisms. Nat. Rev. Neurosci..

[12] Clark A. (2013). Whatever next? Predictive brains, situated agents, and the future of cognitive science. Behav. Brain Sci..

[13] Buccino G., Colagè I., Gobbi N., Bonaccorso G. (2016). Grounding meaning in experience: A broad perspective on embodied language. Neurosci. Biobehav. Rev..

[14] Barsalou L.W. (2008). Grounded cognition. Annu. Rev. Psychol..

[15] Nweke H.F., Teh Y.W., Al-Garadi M.A., Alo U.R. (2018). Deep learning algorithms for human activity recognition using mobile and wearable sensor networks: State of the art and research challenges. Expert Syst. Appl..

[16] Hinton G., Vinyals O., Dean J. Distilling the knowledge in a neural network. Proceedings of the NeurIPS Deep Learning and Representation Learning Workshop.

[17] Gou J., Yu B., Maybank S.J., Tao D. (2021). Knowledge distillation: A survey. Int. J. Comput. Vis..

[18] Romero A., Ballas N., Kahou S.E., Chassang A., Gatta C., Bengio Y. Fitnets: Hints for thin deep nets. Proceedings of the International Conference on Learning Representations.

[19] Park W., Kim D., Lu Y., Cho M. (2019). Relational knowledge distillation. Proceedings of the IEEE/CVF Conference on Computer Vision and Pattern Recognition.

[20] You S., Xu C., Xu C., Tao D. (2017). Learning from multiple teacher networks. Proceedings of the ACM SIGKDD International Conference on Knowledge Discovery and Data Mining.

[21] Zhang H., Chen D., Wang C. (2022). Confidence-aware multi-teacher knowledge distillation. Proceedings of the IEEE International Conference on Acoustics, Speech and Signal Processing (ICASSP).

[22] Gupta S., Hoffman J., Malik J. (2016). Cross modal distillation for supervision transfer. Proceedings of the IEEE Conference on Computer Vision and Pattern Recognition.

[23] Yuan Z., Yang Z., Ning H., Tang X. (2024). Multiscale knowledge distillation with attention based fusion for robust human activity recognition. Sci. Rep..

[24] Ordóñez F.J., Roggen D. (2016). Deep Convolutional and LSTM Recurrent Neural Networks for Multimodal Wearable Activity Recognition. Sensors.

[25] Ma H., Li W., Zhang X., Gao S., Lu S. (2019). AttnSense: Multi-level Attention Mechanism for Multimodal Human Activity Recognition. Proceedings of the Twenty-Eighth International Joint Conference on Artificial Intelligence (IJCAI).

[26] Kwon H., Abowd G.D., Plötz T. (2021). Complex Deep Neural Networks from Large Scale Virtual IMU Data for Effective Human Activity Recognition Using Wearables. Sensors.

[27] Buciluǎ C., Caruana R., Niculescu-Mizil A. (2006). Model compression. Proceedings of the 12th ACM SIGKDD International Conference on Knowledge Discovery and Data Mining.

[28] Radford A., Kim J.W., Hallacy C., Ramesh A., Goh G., Agarwal S., Sastry G., Askell A., Mishkin P., Clark J. (2021). Learning transferable visual models from natural language supervision. Proceedings of the International Conference on Machine Learning.

[29] Frome A., Corrado G.S., Shlens J., Bengio S., Dean J., Ranzato M., Mikolov T. (2013). DeViSE: A deep visual-semantic embedding model. Proceedings of the Advances in Neural Information Processing Systems.

[30] Lüddecke T., Ecker A. Image Segmentation Using Text and Image Prompts. Proceedings of the IEEE/CVF Conference on Computer Vision and Pattern Recognition (CVPR).

[31] Rombach R., Blattmann A., Lorenz D., Esser P., Ommer B. High-Resolution Image Synthesis with Latent Diffusion Models. Proceedings of the IEEE/CVF Conference on Computer Vision and Pattern Recognition (CVPR).

[32] Ferrara E. (2024). Large Language Models for Wearable Sensor-Based Human Activity Recognition, Health Monitoring, and Behavioral Modeling: A Survey of Early Trends, Datasets, and Challenges. Sensors.

[33] Moon S., Madotto A., Lin Z., Saraf A., Bearman A., Damavandi B. (2023). IMU2CLIP: Language-grounded Motion Sensor Translation with Multimodal Contrastive Learning. Proceedings of the Findings of the Association for Computational Linguistics: EMNLP 2023.

[34] Haresamudram H., Beedu A., Rabbi M., Saha S., Essa I., Ploetz T. (2025). Limitations in Employing Natural Language Supervision for Sensor-Based Human Activity Recognition—And Ways to Overcome Them. Proceedings of the AAAI Conference on Artificial Intelligence.

[35] Cheraghian A., Rahman S., Fang P., Roy S.K., Petersson L., Harandi M. (2021). Semantic-Aware Knowledge Distillation for Few-Shot Class-Incremental Learning. Proceedings of the IEEE/CVF Conference on Computer Vision and Pattern Recognition (CVPR).

[36] Li M., Zhang L., Zhu M., Huang Z., Yu G., Fan J., Chen T. (2024). Lightweight Model Pre-Training via Language Guided Knowledge Distillation. IEEE Trans. Multimed..

[37] Vaswani A., Shazeer N., Parmar N., Uszkoreit J., Jones L., Gomez A.N., Kaiser L., Polosukhin I. (2017). Attention Is All You Need. Proceedings of the Advances in Neural Information Processing Systems.

[38] He K., Zhang X., Ren S., Sun J. (2016). Deep Residual Learning for Image Recognition. Proceedings of the IEEE Conference on Computer Vision and Pattern Recognition.

[39] Wang Q., Lohit S., Toledo M.J., Buman M.P., Turaga P. (2016). A statistical estimation framework for energy expenditure of physical activities from a wrist-worn accelerometer. Proceedings of the Annual International Conference of the IEEE Engineering in Medicine and Biology Society.

[40] Reiss A., Stricker D. (2012). Introducing a new benchmarked dataset for activity monitoring. Proceedings of the International Symposium on Wearable Computers.

[41] Jeon E.S., Choi H., Shukla A., Wang Y., Buman M.P., Turaga P. (2022). Topological Knowledge Distillation for Wearable Sensor Data. Proceedings of the Asilomar Conference on Signals, Systems, and Computers.

[42] Jeon E.S., Som A., Shukla A., Hasanaj K., Buman M.P., Turaga P. (2022). Role of Data Augmentation Strategies in Knowledge Distillation for Wearable Sensor Data. IEEE Internet Things J..

[43] Jordao A., Nazare A.C., Sena J., Schwartz W.R. (2018). Human activity recognition based on wearable sensor data: A standardization of the state-of-the-art. arXiv.

[44] Zagoruyko S., Komodakis N. Wide Residual Networks. Proceedings of the British Machine Vision Conference (BMVC).

[45] Cho J.H., Hariharan B. (2019). On the Efficacy of Knowledge Distillation. Proceedings of the IEEE/CVF International Conference on Computer Vision (ICCV).

[46] OpenAI (2026). GPT-5.4 Thinking System Card. https://openai.com/index/gpt-5-4-thinking-system-card/.

[47] Nussbaum Z., Morris J.X., Mulyar A., Duderstadt B. (2025). Nomic Embed: Training a Reproducible Long Context Text Embedder. arXiv.

[48] Zagoruyko S., Komodakis N. (2017). Paying more attention to attention: Improving the performance of convolutional neural networks via attention transfer. Proceedings of the International Conference on Learning Representations.

[49] Tung F., Mori G. (2019). Similarity-preserving knowledge distillation. Proceedings of the IEEE International Conference on Computer Vision.

[50] Chen D., Mei J.P., Zhang H., Wang C., Feng Y., Chen C. (2022). Knowledge distillation with the reused teacher classifier. Proceedings of the IEEE/CVF Conference on Computer Vision and Pattern Recognition.

[51] Huang T., You S., Wang F., Qian C., Xu C. (2022). Knowledge distillation from a stronger teacher. Adv. Neural Inf. Process. Syst..

[52] Miles R., Mikolajczyk K. (2024). Understanding the role of the projector in knowledge distillation. Proceedings of the AAAI Conference on Artificial Intelligence (AAAI).

[53] van der Maaten L., Hinton G. (2008). Visualizing Data using t-SNE. J. Mach. Learn. Res..

[54] Rosenberg A., Hirschberg J. (2007). V-measure: A conditional entropy-based external cluster evaluation measure. Proceedings of the Joint Conference on Empirical Methods in Natural Language Processing and Computational Natural Language Learning (EMNLP-CoNLL).

[55] Guo C., Pleiss G., Sun Y., Weinberger K.Q. (2017). On calibration of modern neural networks. Proceedings of the International Conference on Machine Learning.

[56] Minderer M., Djolonga J., Romijnders R., Hubis F., Zhai X., Houlsby N., Tran D., Lucic M. (2021). Revisiting the Calibration of Modern Neural Networks. Proceedings of the Advances in Neural Information Processing Systems.

[57] Wang S., Wan Q., Gao J., Zeng Z. (2025). Language-Driven Multi-Label Zero-Shot Learning with Semantic Granularity. Proceedings of the IEEE/CVF International Conference on Computer Vision (ICCV).

[58] Ding T., Li W., Miao Z., Pfister H. (2025). Tree of Attributes Prompt Learning for Vision-Language Models. Proceedings of the International Conference on Learning Representations (ICLR).

[59] Saha O., Van Horn G., Maji S. (2024). Improved Zero-Shot Classification by Adapting VLMs with Text Descriptions. Proceedings of the IEEE/CVF Conference on Computer Vision and Pattern Recognition (CVPR).

[60] Jeon E.S., Choi H., Buman M.P., Turaga P. (2024). Role of Mixup in Topological Persistence-Based Knowledge Distillation for Wearable Sensor Data. IEEE Sens. J..

[61] Gao Y., He Y., Li X., Zhao B., Lin H., Liang Y., Zhong J., Zhang H., Wang J., Zeng Y. (2024). An empirical study on low gpu utilization of deep learning jobs. Proceedings of the IEEE/ACM International Conference on Software Engineering.

[62] Zhang H., Huang J. (2025). Challenging GPU Dominance: When CPUs Outperform for On-Device LLM Inference. arXiv.

